# Histological appearance of topical hemostatic agents and materials in neuropathology

**DOI:** 10.1093/jnen/nlaf032

**Published:** 2025-04-16

**Authors:** Barbara Verbraeken, Tomas Menovsky, Rabih Aboukais, Martin Lammens

**Affiliations:** Department of Pathology, Antwerp University Hospital (UZA), Antwerp, Belgium; Faculty of Medicine and Health Sciences, University of Antwerp (UA), Antwerp, Belgium; Faculty of Medicine and Health Sciences, University of Antwerp (UA), Antwerp, Belgium; Department of Neurosurgery, Antwerp University Hospital (UZA), Antwerp, Belgium; Department of Neurosurgery, Lille University Hospital, Lille, France; Department of Pathology, Antwerp University Hospital (UZA), Antwerp, Belgium; Faculty of Medicine and Health Sciences, University of Antwerp (UA), Antwerp, Belgium

**Keywords:** foreign body, hemostatic agents, histology, neurosurgery, pseudotumor progression, surgical materials

## Abstract

Hemostatic agents and other foreign materials are frequently encountered in neuropathology samples. Recognizing these materials is crucial for accurate diagnosis and in the context of adverse events. This article provides an overview of macroscopic and microscopic characteristics of commonly used hemostatic agents and materials in neurosurgery. Samples of sterile hemostatic agents and retrospectively collected pathology slides were examined. Routine histopathological processing, special stains, and polarized light microscopy were utilized to document the appearance of these materials. A total of 22 hemostatic agents and 9 artifacts and foreign bodies were analyzed. Distinct macroscopic and microscopic properties, as well as effects of tissue processing, were documented. Recognizing hemostatic agents and materials is largely dependent on their main constituents. A constituent-based approach for identification of these materials is presented for the practicing neuropathologist.

## INTRODUCTION

Remnants of hemostatic agents and materials used during neurosurgery are quite frequently present in neuropathology samples. An accurate neuropathological diagnoses can be challenging without at least some background knowledge of such materials. Being able to identify hemostatic agents on microscopy is not just a party trick as it can be of real importance in cases of adverse events or in cases with pseudotumor progression due to granuloma formation.^1–4^ Only limited data on the appearance of hemostatic agents are available in the literature.^5–7^ This paper summarizes the macroscopic and microscopic appearances of commonly used hemostatic agents, as well as some materials and tissue artifacts that may be encountered in neuropathology.

Agents used for intraoperative hemostasis during neurosurgical procedures are frequently left in situ. These hemostats are primarily used for stopping oozing bleeding. They are left in place to avoid disturbing the newly formed clot, risking a rebleed. Hemostatic agents from prior surgical procedures may thus be present in neuropathology samples, even in a partially degraded state.

The most widely used hemostatic agents in neurosurgery consist of regenerated natural fibers (mainly cellulose) and substances of animal origin, processed to eliminate pollutants and human pathogens. Hemostats of animal origin include bone wax (Ethicon, Johnson & Johnson, Raritan, NJ), which is primarily composed of beeswax, collagen and its derivatives, and proteins with specific thrombogenic properties such as aprotinin, fibrinogen, and thrombin.

Collagen and gelatin are widely employed for neurosurgical hemostasis and are available in a variety of shapes. TachoSil (Corza Medical GmbH, Düsseldorf, Germany), and Floseal (Baxter International, Inc., Hayward, CA), and SurgiFlo (Ethicon, Johnson & Johnson) are hemostatic agents that consist of a combination of a collagen-based agent and human or recombinant thrombin, to increase their hemostatic potency.

Oxidized regenerated cellulose (ORC), a regenerated natural polymer, was developed in the Kodak laboratories in the 1940s.^8^^,^^9^ ORC now comes in an array of different knits, weaves, and powders, produced by multiple manufacturers. These cellulose fibers have been used for neurosurgical hemostasis since at least 1943,^10^ and have steadily been improved in terms of purity and structure.

Some hemostats are derived from pure vegetal material. This includes the powdered microporous polysaccharide hemospheres (Arista AH, BD—Becton, Dickinson and Company, Franklin Lakes, NJ), which consist of polysaccharide particles made from purified potato starch. Natural cotton from surgical gauze may be left in situ unintentionally. The smaller neurosurgical patties or “cottonoids” are usually not made of natural cotton but composed of rayon or polyester fibers.

Entirely synthetic polymers are rarely used in neurosurgery and are virtually non-existent for neurosurgical hemostasis. Synthetic, man-made materials are implanted only in specific instances. For example, Teflon (polytetrafluoroethylene or PTFE) is used for microvascular decompression surgery.^11^ Polysiloxane (silicone) is present in some synthetic dura replacements (eg, Biomesh Cousin Biotech, Wervicq-Sud, France). Ostene (Baxter International, Inc.) was developed as a synthetic alternative to bone wax and consists of an alkylene oxide copolymer.^12^ Polyvinyl alcohol (PVA) sponges,^13^ synthetic sutures, polyetheretherketone plastic, and synthetic glues made of polyolefins (polyethylene glycol) and cyanoacrylate all have specific uses in neurosurgery. Mineral and inorganic fibers in neuropathology always represent unintentional foreign bodies.

## METHODS

Samples of sterile (unused) hemostatic agents and retrospectively collected microscopy slides from our institution were used to establish an accurate representation of each hemostatic agent. The slides were of hemostatic agents retrieved during neurosurgical reoperation. Indications for reoperation included surgical complications, redo-surgeries and staged procedures. At our institution, hemostatic agents and materials encountered during neurosurgical reoperations are regularly sent for histopathological examination. The type of agent sent for examination was indicated on the histopathology request form. In addition, samples and slides of other materials and artifacts encountered in neurosurgical pathology were reviewed. All materials were received from the Department of Neurosurgery at Antwerp University Hospital, without financial support or sponsorship from the manufacturer.

Samples underwent routine histopathological processing, including formalin fixation, paraffin embedding, microtomy, and hematoxylin and eosin (H&E) staining using an automated stainer. The unused hemostatic agents were soaked with normal saline and 4% buffered formalin for up to 48 hours prior to processing. Some of the samples retrieved retrospectively had been stained using both conventional and special stains (eg, elastin) and/or immunohistochemistry. Special stains and immunohistochemistry slides were reviewed to detect any notable staining patterns. An experienced investigator (B.V.) examined all samples using standard light microscopy and polarized light microscopy.

Microscopy slides were photographed using the Philips IMS system for scanned slides with the Philips whole slide imaging (WSI) scanning system. When slides could not be scanned, when WSI was blurry, or when polarization was required, a microscope-mounted phone camera was used (Apple iPhone 11).

Informed consent was present for all samples collected from human subjects. Ethics approval for the analysis of unused hemostatic agents and materials was waived. Ethics approval for the retrospective review of samples retrieved from hemostatic agents and materials during reoperations that were previously sent for histological examination was granted by the local ethics committee of Antwerp University Hospital (Edegem, Belgium) under the reference number 000850.

## RESULTS

This study includes descriptions of 22 hemostatic agents and 9 artifacts and materials. This includes 19 samples of unused hemostatic agents, and historical samples of 20 different agents and materials previously retrieved during neurosurgical reoperation.

Table 1 lists the hemostatic agents and materials examined. The major macro- and microscopic characteristics of the different hemostatic agents are illustrated in the figures. Materials and artifacts that may be confused with hemostatic agents are briefly described. Figures 1-10 depict hemostatic agents organized by their main constituent while Figures 11-14 depict artifacts and materials.

**Figure 1.**
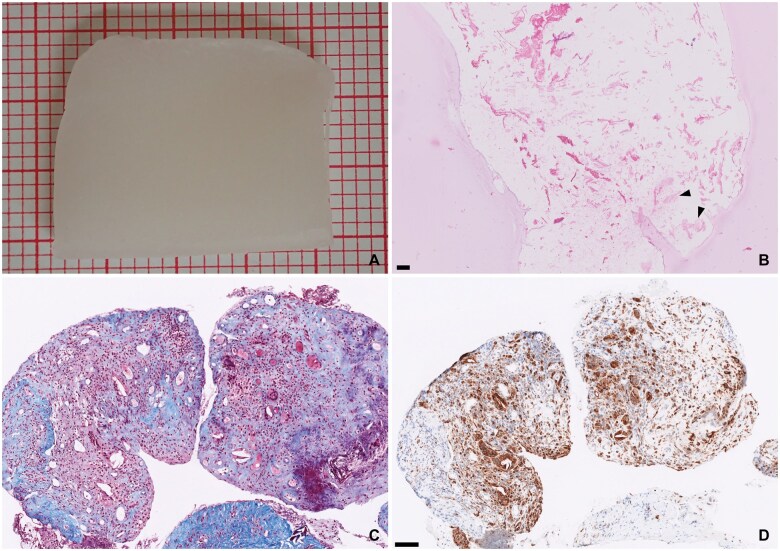
**Bone wax.** Bone wax is a semi-soft wax, often molded into small spheres and then smeared (A, on red millimeter paper). Bone wax appears as gaps and empty spaces when processed for routine histopathology. Although the spaces are mostly empty, sometimes bone wax intermixed with fibrin may be seen, giving the wax a slight eosinophilic tint (B, arrowheads). Gaps may also be smaller when breakdown is taking place (C, Masson-Trichrome stain). CD68 immunohistochemistry shows multinucleate giant cells surrounding gaps in the tissue block (D). Scale bar = 100 µm.

**Figure 2.**
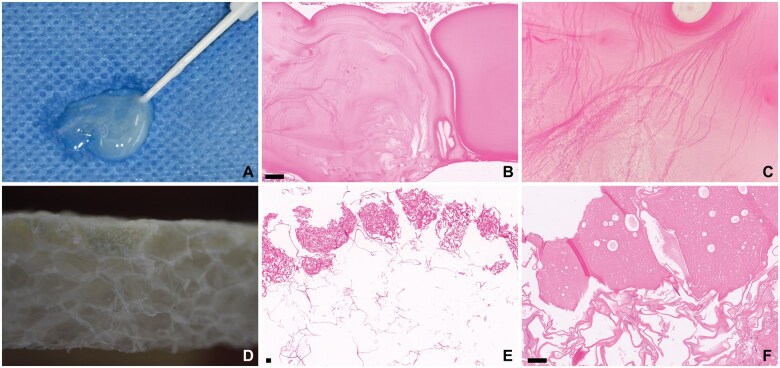
**Fibrin sealant and TachoSil.** (A-C) Fibrin sealant. Fibrin glue (macroscopic image in A) has a solid, bright eosinophilic and slightly lamellated appearance on H&E with rare, entrapped air bubbles (B, C). (D-F) TachoSil. TachoSil is a mixture of human fibrinogen and human thrombin on an equine collagen sponge. The fibrinogen-thrombin coating forms the dense surface (D, top surface with yellow hue on the macroscopic image), and a porous white side (D, porous bottom). This appearance is readily identifiable microscopically (E, F). As TachoSil is compressed, the porous side appears as stacked lamellae and the dense coating becomes more compact (F). The compact coating retains multiple entrapped air bubbles (F). Scale bar = 100 µm.

**Figure 3.**
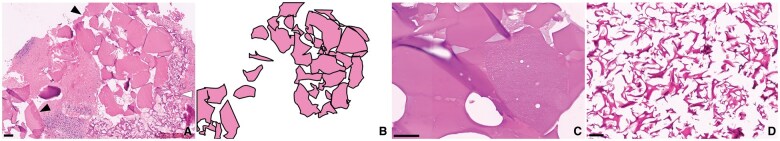
**Floseal and Surgiflo.** Floseal is easily distinguished from other gelatin-based agents because it forms large clumps of amorphous, deep eosinophilic material (A, black arrowheads & B, outlines of the material seen in panel A). Compare this to the much smaller gelatin sponge (A, white arrowhead). The contours of the gelatin particles are smooth and air bubbles within the clumps are rare. There is no lamination (C). Surgiflo particles are much smaller and appear indistinguishable from gelatin sponge (D, compare with gelatin sponges depicted in Figure 5). Scale bar = 100 µm.

**Figure 4.**
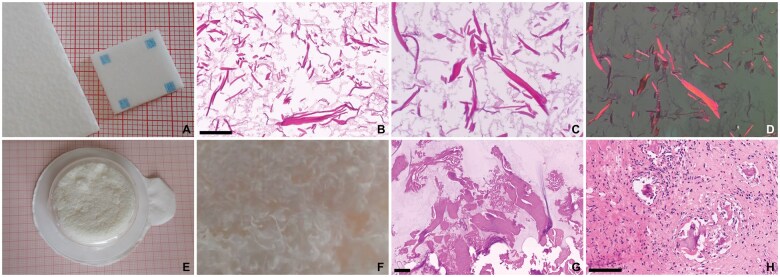
**Collagen-based hemostatic agents.** (A-D) Hemopatch. This collagen-based agent is coated with a layer of synthetic material to improve tissue adhesion (A). The amphophilic bands of collagen form loose bundles or single strips, and the background is made up of porous basophilic material (B, C). Characteristic orange-pink birefringence is present (D). (E-H) Avitene, a microfibrillar collagen hemostat, consists of a powdery, fibrillar substance that forms strands and clots when wet (E, magnified in F). It consists of strips of basophilic or amphophilic material that are clumped together and feathered at the ends (G). The collagen clumps may leave empty gaps and spaces that resemble bone wax, but an area of retained collagen strands or clumps can usually be found (H). Scale bar = 100 µm, red millimeter paper.

**Figure 5.**
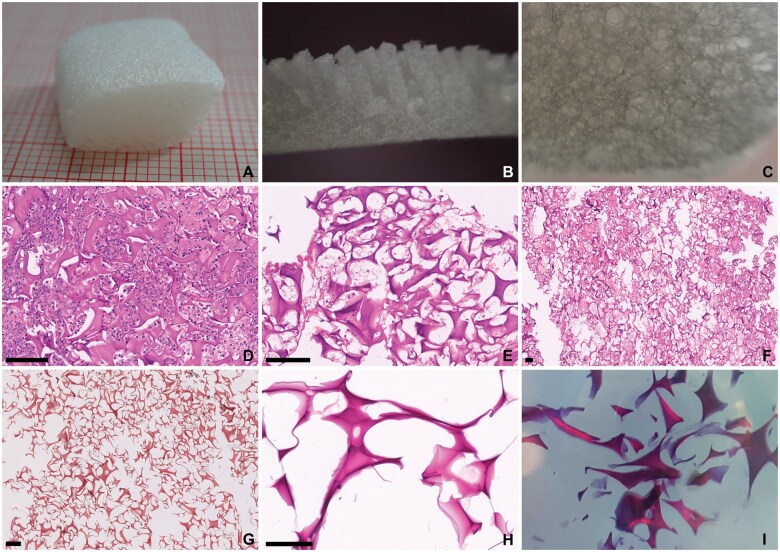
**Hemostatic gelatin sponges.** Gelatin-foams are sponges with a white, satin gloss. They come in a variety of different sizes and cuts (A-C). Microscopically, they share a similar trabeculated structure of triradiate and tetraradiate particles with smooth outlines (D-I). In tissue, the pores may be filled with blood, inflammatory cells, and even tumor cells (D). The particles were negative on elastin stain (G). There is slight orange-pink birefringence on polarized light microscopy (I). (A, C-D, G, I) Spongostan. (B) TenaTac. (E) CuraSpon. (F, H) Gelfoam. Scale bar = 100 µm, red millimeter paper.

**Figure 6.**
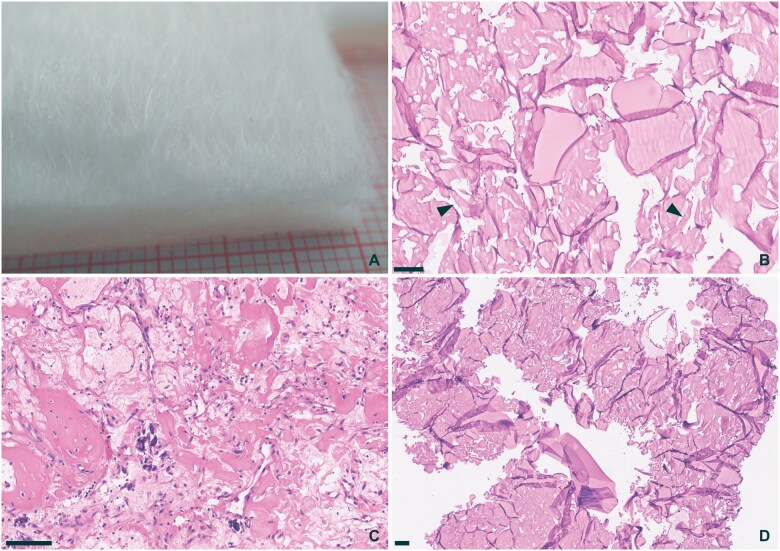
**Tuft-It and Gelita-Spon powder.** Tuft-It may resemble Floseal under the microscope, but contains thinner fibrillar structures (A and B, arrowheads). These are usually not identifiable when they are encountered in neuropathology samples (C). The powder mixture of Gelita-Spon Powder (Geli Putty) looks nearly identical (D). Scale bar = 100 µm, red millimeter paper.

**Figure 7.**
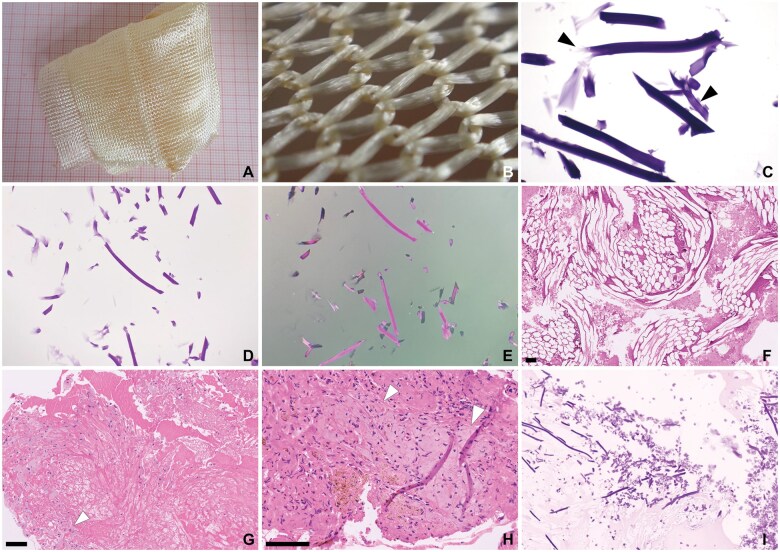
**Regular and knit ORC.** (A-H) Surgicel Regular. Cellulose fibers in a loosely woven fashion (A, red millimeter paper). The thread is composed of bundles of cellulose fibers (B), that fray when cut. When preserved after processing, ORC fibers are basophilic on microscopy. They are striated along their longitudinal axis, with fraying of the cut ends (C, black arrowheads). The fibers are purple birefringent (D, E). Most often, ORC fibers have dissolved during processing of tissue for microscopy (F, G). The outlines of the fibers and some remnants may be seen, surrounded by fibrin and blood (G). Breakdown of ORC by macrophages may result in groups of macrophages filled with basophilic material (G, white arrowhead). These groups of macrophages may mimic a neoplastic process (H, white arrowheads). (I**)** Surgicel Nu-Knit. The more intricately woven Nu-Knit is not readily distinguishable from the regular weave on light microscopy. Scale bar = 100 µm.

**Figure 8.**
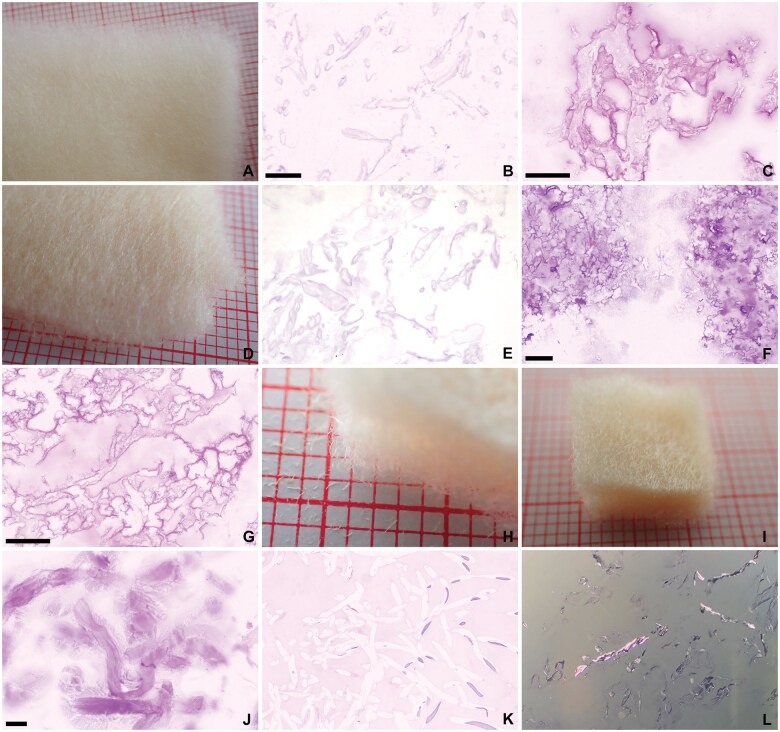
**Fibrillar types of ORC.** (A-C) Stypcel. The fibrillar texture of ORC fibers on macroscopy is largely lost on microscopy. (D-F) Traumastem fibrillar. Only softly basophilic remnants remain. (G) Traumastem non-woven. Fibrillar ORC remnants usually have a deeply basophilic and irregular outline, and a light and cloudy center. (H-L) Surgicel fibrillar. Agar embedding of fibrillar ORC preserves some of the fibrils (J). Partial dissolution of the ORC fibers may still be seen, with remnants of basophilic fibers on the right of the image (K). Purple birefringence of the ORC fibrils (L). Scale bar = 100 µm, red millimeter paper.

**Figure 9.**
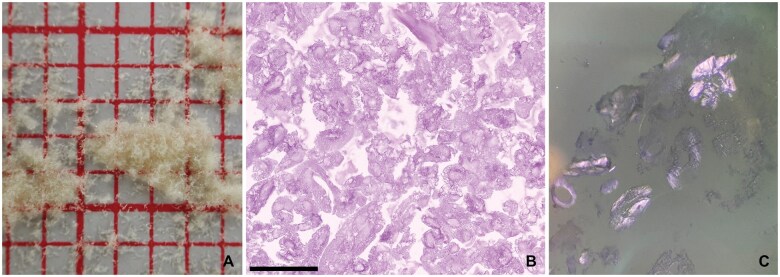
**Gelita-Cel Ca powder.** This cellulose-calcium powder mixture consists of ultrafine fibrillar structures (A), that present as incoherent, cloudy, translucent, basophilic dust on microscopy (B). The material is sometimes difficult to see on H&E stain but is clearly birefringent (C). Scale bar = 100 µm, red millimeter paper.

**Figure 10.**
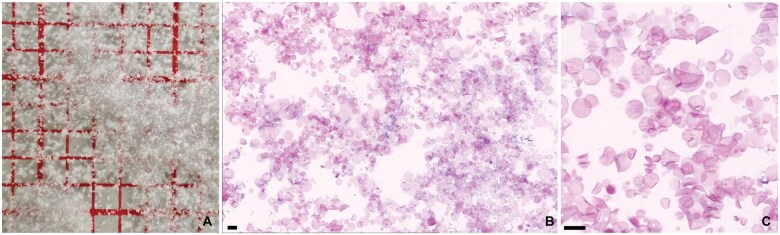
**Arista AH.** A starch-based hemostat that comes as a true-white powder (A). Hemospheres of different sizes are easily identifiable by microscopy (B, C). These eosinophilic spheres have smooth borders and rare entrapped air bubbles (C). Scale bar = 100 µm.

**Figure 11.**
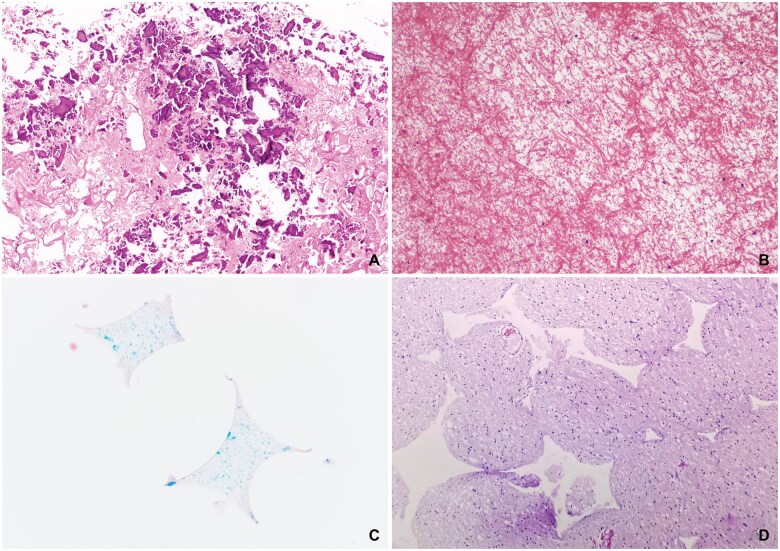
**Artifacts.** Bone fragments are commonly found near hemostatic agents used close to the site of craniotomy. These fragments are usually tiny and are remnants of bone dust or vertebral bone (A). Delicate fibrin strands (diameter of 100-200 nm) are easily distinguished from hemostatic agents or materials, which are usually much larger (B). Fragments of (blue) sponges used for tissue embedding (C) and their resulting artifacts (D) may be seen in neurosurgical samples. These are quite easy to discern from true foreign objects and are not associated with any tissue response (D). Compression of neurosurgical specimens in the tissue cassette (usually due to the use of these embedding sponges) should be avoided.

**Figure 12.**
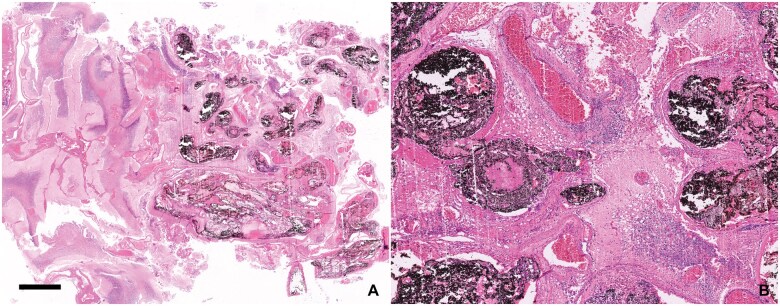
**Onyx embolization material in vascularized tissues.** Preoperative embolization may be evident in samples from vascular lesions (eg, arteriovenous malformations) and highly vascularized tumors (eg, meningiomas). The intravascular residue seen microscopically consists of “casts” of blood vessels, usually slightly displaced on the slide due to the rigidity of the substance on microtomy (A). Bright and dark substances exist for embolization, and the microscopic appearance is highly dependent on the type of agent used. The irregular, deeply black residue resembles carbon or soot pigment (B). Scale bar = 2 µm.

**Figure 13.**
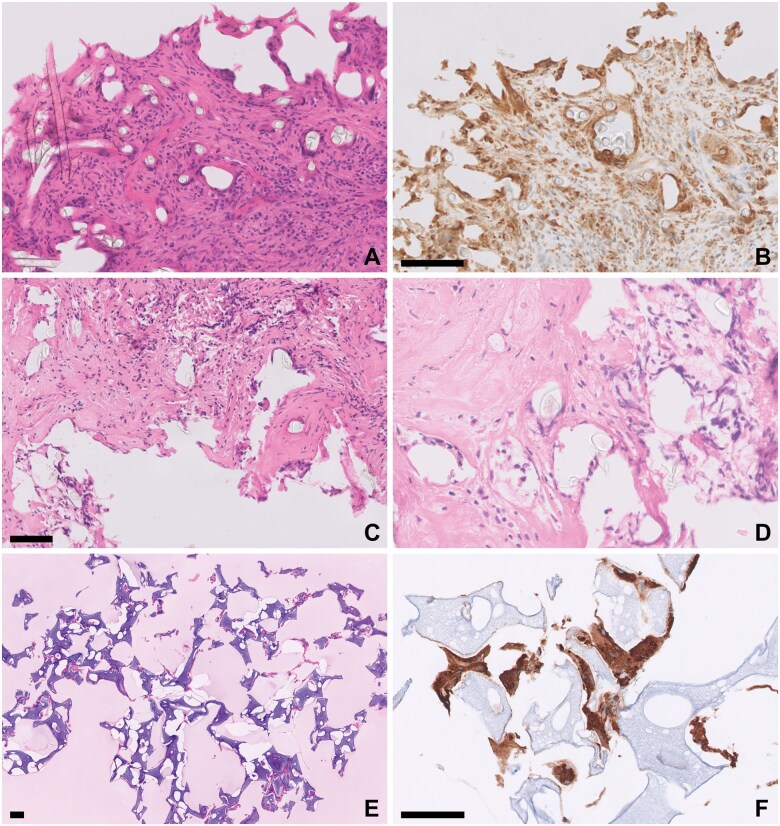
**Synthetic material.** Foreign bodies from duraplasty, microvascular decompression surgery, vessel wrapping or grafting, and artifacts from materials unintentionally implanted into tissue may also be seen. Examples of these include silicone fibers, shredded Teflon felt used in microvascular decompression, and PVA sponges (also microvascular decompression). (A, B) Biomesh dural replacement. Silicone fibers are seen as transparent, uniform, round fibrils with minimal pigmentation (A). CD68 immunohistochemistry shows the silicone fibers within the cytoplasm of multinucleate giant cells (B). (C, D) Teflon. Transparent fibers of shredded Teflon fleece in fibrous tissue (C) and in the cytoplasm of multinucleate giant cells (D). (E, F) PVA sponge. The porous, basophilic material embedded in agar is characteristically vacuolated with slightly irregular borders (E). CD68 immunohistochemical stain again displays multinucleate giant cells surrounding the PVA fragments (F). Scale bar = 100 µm.

**Figure 14.**
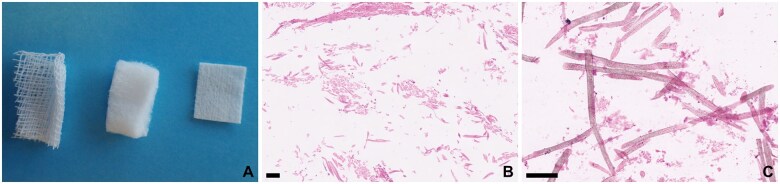
Sterile surgical gauze and neurosurgical patties (also known as “cottonoids”) are infrequently present in neurosurgical slides. These are usually picked up on radiological imaging and are quite easy to distinguish from other materials. (A) Cotton (left) and neurosurgical patties of 2 sizes (center and right). (B) Cotton fibers. Natural cotton has characteristically hollow, tubular fibers with variable diameters. Surgical gauze is usually made from natural cotton, as are some neurosurgical patties. (C) Neurosurgical patties. Most cottonoids are composed of rayon (irregular fibers) or nylon (regular fibers). These have smooth outlines and contain dark speckles of delustering granules. While they may resemble human hair on microscopy, the fibers are generally one-fifth the width of a human hair. Scale bar = 100 µm.

**Table 1.** Featured neurosurgical materials and hemostatic agents.

	Manufacturer	Main component	Unused samples (*X* = analyzed)	Retrieved samples (*N* = count)
**Hemostatic agents**				
Arista AH MPH	BD—Becton, Dickinson and Company	potato starch	X	
Bone Wax	Ethicon, Johnson & Johnson	beeswax	X	9
Avitene MCH	BD—Becton, Dickinson and Company	microfibrillar collagen	X	1
Tisseel Fibrin Sealant	Baxter International Inc.	human fibrinogen, Factor XIII, aprotinin, human thrombin	X	23
Surgiflo Hemostatic Matrix with thrombin	Ethicon, Johnson & Johnson	porcine gelatin and human thrombin	X	
Floseal hemostatic thrombin matrix	Baxter International Inc.	bovine gelatin and human thrombin	X	1
CuraSpon	CuraMedical B.V.	gelatin		1
TenaTac	Selentus Science Limited & CuraMedical B.V.	porcine gelatin	X	
Gelita Tuft-It	Gelita Medical GmbH	porcine gelatin		6
Gelita-Spon Powder (formerly: Geli Putty)	Gelita Medical GmbH	porcine gelatin		2
Gelita-Spon	Gelita Medical GmbH	porcine gelatin	X^a^	3^a^
Spongostan	Ethicon, Johnson & Johnson	porcine gelatin	X	28
Gelfoam	Pfizer Inc.	porcine skin gelatin	X	10
Gelita-Cel Ca Powder	Gelita Medical GmbH	ORC, hydrogen calcium salt	X	1
Stypcel	Medprin Biotech GmbH	ORC	X	
Surgicel Nu-Knit	Ethicon, Johnson & Johnson	ORC	X	
Surgicel Fibrillar	Ethicon, Johnson & Johnson	ORC	X	1
Surgicel Regular	Ethicon, Johnson & Johnson	ORC	X	22
Traumastem fibrillar	Baxter International Inc.	ORC	X	
Traumastem non-woven	Baxter International Inc.	ORC	X	
TachoSil fibrin sealant patch	Corza Medical GmbH	human fibrinogen and human thrombin on a collagen sponge	X	39
Hemopatch	Baxter International Inc.	bovine collagen, coating layer of NHS-PEG	X	
**Materials/Foreign Objects**				
Biomesh Patch N3 L Neurological patches	Cousin Biotech	silicone (polyethylene terephthalate + dimethyl siloxane)		8
Cotton (Surgical Gauze)		cotton	X	3
Neurosurgical patty (cottonoid)		rayon (cellulose), others: polyester, cotton	X	
Teflon		PTFE		2
Onyx liquid embolic system (LES)	Medtronic	ethylene vinyl-alcohol copolymer, dimethyl-sulfoxide and micronized tantalum powder		2
PVA		PVA		2

List of analyzed materials and topical hemostatic agents; unused or retrieved during neurosurgical reoperation.

a Not depicted in figures.

Abbreviations: MCH, microfibrillar collagen hemostat; MPH, microporous polysaccharide hemospheres; NHS-PEG, pentaerythritol polyethylene glycol ether tetra-succinimidyl glutarate; ORC, oxidized regenerated cellulose; PTFE, polytetrafluoroethylene; PVA, polyvinyl alcohol.

### Bone wax

Bone wax (ie, beeswax) is used to stop bleeding from damaged bony surfaces. It shares most of its physical properties with the paraffin used for tissue embedding in routine processing of samples for pathology and is, therefore, often indistinguishable from the paraffin of the sectioned block (Figure 1). A vague pink hue may be noted in the otherwise empty spaces where the bone wax was present, especially if the bone wax had time to get intermixed with blood and fibrin. Sometimes, overt foreign body reactions are seen surrounding the empty spaces (Figure 1C and D).

### Fibrinogen and thrombin

Fibrin sealant, such as Tisseel (formerly Tissucol; Baxter International, Inc.), and TachoSil are hemostatic agents containing both fibrinogen and human thrombin. These agents are often found in samples obtained in the proximity of the dura, because of their frequent application as dural sealants. Tisseel is a translucent, off-white gel that comes in a prefilled syringe, and hardens into a white, rubbery consistency after application. TachoSil is a sponge made from equine collagen, coated with a thin layer of the fibrinogen-thrombin mixture on one side (Figure 2).

Other flowable agents containing a mixture of bovine or porcine gelatin and thrombin are Surgiflo Hemostatic Matrix with thrombin (Ethicon, Johnson & Johnson) and Floseal (Baxter International Inc.). These are considered more potent in terms of hemostasis and do not harden upon application. With gelatin as their main constituent, these agents share their microscopic characteristics with other gelatin- and collagen-based agents (Figure 3).

### Collagen and gelatin-based agents

Aside from TachoSil (Corza Medical GmbH), other collagen-based agents like Hemopatch (Baxter International, Inc.) and Avitene Microfibrillar Collagen Hemostat (BD—Becton, Dickinson and Company) have been approved for neurosurgical use (Figure 4). Avitene resembles the material of gelatin sponges described below, the compacted microfibrillar collagen forms larger clots and bands of amorphous material; as previously described by other authors.^14^^,^^15^

We found the remnants of Avitene to be mostly basophilic on H&E, as depicted in Perry and Brat.^5^ Larger remnants of this microfibrillar collagen hemostat have also been described as deeply eosinophilic and have been known to cause granulomatous reactions mimicking tumor recurrence.^14^^,^^15^

Hemopatch is made from bovine collagen and is covered with a coating layer of NHS-PEG (pentaerythritol polyethylene glycol ether tetra-succinimidyl glutarate) for increased tissue adhesion. Microscopic sections of unused Hemopatch consist of amphophilic fibrils with a characteristic orange-pink hue on polarized light microscopy. This birefringence appears similar to the birefringence of gelatin-based agents but was slightly more pronounced.

Gelatin-based hemostats, aside from the flowables already described, are mostly gelatin sponges. Brand names include Gelita-Spon (Gelita Medical GmbH, Eberbach, Baden, Germany), Spongostan (Ethicon, Johnson & Johnson), Gelfoam (Pfizer Inc., New York, NY), CuraSpon (CuraMedical B.V., Assendelft, The Netherlands), TenaTac (Selentus Science, Ltd and CuraMedical B.V.), and others.

These materials resemble one another, and they cannot be reliably distinguished by microscopic examination. They consist of a porous, reticular structure of smooth, basophilic particles. These particles are triradiate and tetraradiate, sometimes with a small, entrapped air bubble. The gelatin is deeply basophilic on H&E and may be found to have a slight orange-pink birefringence (Figure 5). We were unable to reproduce the intense ink-black staining of gelatin particles with Verhoeff's Van Gieson staining (Figure 5G), as previously described by other authors.^7^

Gelatin powders such as Gelita-Spon Powder (formerly: Geli Putty; Gelita Medical GmbH) and fleece from porcine gelatin (Tuft-It, Gelita Medical GmbH) are less frequently used. These gelatin fibrils and particles form larger clumps or strands upon application. Otherwise, they share the characteristic appearance of other gelatin-based agents (Figure 6).

### Plant-based agents

ORC hemostats have gone through multiple modifications. They now come in a variety of weaves, knits, and fleeces, for example, Stypcel (Medprin Biotech GmbH, Frankfurt am Main, Germany), Surgicel Regular, Surgicel Fibrillar, Surgicel Nu-Knit (Ethicon, Johnson & Johnson), Traumastem Fibrillar, Traumastem Non-Woven, and Traumastem Woven (Baxter International, Inc.) (Figures 7 and 8). Some are available as powders, for example, Gelita-Cel Calcium Powder (Gelita Medical GmbH, Eberbach) (Figure 9).

Histologically, preserved ORC fibers are basophilic and show longitudinal striations, which are also evident on fray ends of fibers (Figure 7C). Powders and fleece from ORC are basophilic and may leave only a light basophilic hue, which may be difficult to discern on H&E but easily identified on polarized light microscopy (Figures 8 and 9).

One notable observation made was that in some cases ORC fibers dissolved during routine histopathology processing. Dissolution of ORC fibers was found to be inconsistent. In some cases, ORC fibers were preserved after processing, while in other samples pieces of unused ORC were macroscopically unrecognizable after submersion in natural-buffered formalin 4% for at least 24 hours. To overcome this phenomenon, agents were embedded in agar prior to fixation, enabling further processing for microscopic examination. ORC fibers in tissue samples had largely dissolved during histological processing. They were still identifiable by the outlines they left behind. If no surrounding tissue was present, they were usually still visible as empty streaks surrounded by fibrin. Remnants of basophilic material within the cytoplasm of macrophages were also seen.

Another plant-based hemostatic agent is the polysaccharide powder Arista AH (BD—Becton, Dickinson and Company). This is one of the few natural plant-derived agents approved for neurosurgical use. This hemostat, made from purified potato starch, consists of so-called microporous polysaccharide hemospheres. This white powdery substance is readily identifiable on light microscopy (Figure 10).

### Artifacts and materials

Artifacts that may be confused with hemostatic agents include bone fragments, from dust created during craniotomy, and fibrin strands (Figure 11A and B). Neither resembles any of the foreign materials used in neurosurgery, making them easy to distinguish once one is familiar with neurosurgical materials. Another artefact commonly encountered in slides are remnants of the embedding sponges and their characteristic tissue impressions. Both considerably differ from any of the neurosurgical implants (Figure 11C and D).

Some of the synthetic materials used in neurosurgery are depicted in Figures 12 and 13. Note the unmistakable foreign body reactions elicited by many of the implanted materials (Figure 13). Cotton and neurosurgical patties may rarely be left in situ unintentionally, causing the iatrogenic complication called “gossypiboma” (from “gossypium” in Latin or “γοσσύπιον” in ancient Greek = cotton). One sample of surgical gauze sent for histology was available, while only unused samples of neurosurgical patties were available for examination (Figure 14).

Cotton can be recognized by a primary and secondary wall around a narrow, tubular lumen.^16^ Because of their natural origin, cotton fibers have variable fiber thickness.

Neurosurgical patties or “cottonoids,” which are specifically designed for microneurosurgery, are smaller and thinner compared to standard surgical gauze. These patties are manufactured to be less prone to losing fibers upon removal. Patties are usually composed of semi-natural or synthetic fibers such as rayon and polyester.^17^ Rayon and cellulose fibers have irregular cross-sections and striations running across the length of the fibrils. Polyamide, polyester, and nylon fibers are mostly smooth, straight, and circular on cross-section, with uniform diameters. Detailed methods for exact fiber identification can be found elsewhere.^16^

## DISCUSSION

This paper covers the macroscopic and microscopic appearances of commonly used neurosurgical hemostats and materials that may be encountered in neuropathology. Sub-classification of a specific type or brand of material based on its histological properties (eg, pore size, fiber thickness), was found to be unreliable. Although one might expect that hemostatic agents could be identifiable by their knit or weave patterns or pore size, this belief did not hold true in practice. For example, Spongostan and Gelfoam, both gelatin-foam sponges, could not be reliably differentiated based on their microscopic appearances.

In some instances, only gaps and holes in the tissue block may point to the presence of a (partially) dissolved hemostatic agent. In our experience, this was the case for samples of bone wax, ORC, and Avitene. Identification of remnants in some of the gaps (Avitene) or inside the cytoplasm of foamy macrophages (ORC) may be helpful. Localized fibrosis in the presence of multinucleate giant cells surrounding empty spaces should prompt consideration of the presence of a hemostat-remnant. Powders and fibrillar variants of ORC may also leave only a faint, basophilic hue on H&E. In these instances, birefringence on polarized light microscopy can be helpful.

We found Surgicel and other ORC derivatives to sporadically dissolve after fixation in 4% buffered formalin or after routine processing. Dissolution of cellulose samples in formaldehyde was inconsistent and could not be explained by differences in tissue handling, storage, processing, or paraffin embedding. Some agents are known solvents for cellulose fibers (eg, ammonium salt, aqueous alkali solutions, cuprammonium hydroxide), but none of these known solvents were used during processing of the samples studied herein. Contradictorily, formaldehyde is applied in the textile industry to strengthen the cross-links between cellulose fibers.^18^ Nevertheless, partial hydrolysis of cellulose fibers over time due to a combination of factors might explain why fibers were sometimes undetectable in the liquid formalin, without having truly dissolved. Processing of hemostatic agents for microscopic examination should include agar-embedding or enveloping of samples in thin paper whenever feasible. Prolonged periods of storage in formalin should be avoided.

### Limitations

A selection of hemostatic agents was included based on availability and frequency of use. Only a limited number of artifacts are featured. Certain synthetic materials were not discussed, including synthetic tissue-glue and Ostene (Baxter International, Inc.), a synthetic alternative to bone wax. Likewise, neuropathology samples may contain hemostatic agents or materials that are not primarily used in neurosurgery. These include iatrogenic emboli of hemostats or coating agents from intravascular devices used in cardiac or vascular procedures.^19–23^ The histological appearance of such emboli entering the cerebrovascular system is demonstrated in previous publications.^19^^,^^20^^,^^24^

### Key observations

Artifacts are generally easy to differentiate from hemostatic agents and materials due to their distinct appearances. Entirely synthetic materials are mostly found intact, surrounded by marked foreign body giant cell reaction and fibrosis. Identification of a hemostatic agent primarily relies on its main constituent, as this correlates with its microscopic appearance. While neurosurgical hemostatic agents are traditionally categorized by their mode of action, this approach is less relevant for the practicing pathologist. Categorizing different hemostatic agents and materials by their main constituents instead provides a better framework for understanding which agents may have similar microscopic appearances. A system loosely based on classifications such as those used in garment manufacturing to categorize textile fibers is probably most helpful. In general, natural materials consist of more variably sized particles or fibers, while machine-made materials are more regularly sized.

Based on our experience and the data from the scientific literature, all currently available hemostatic agents have been associated with at least some degree of inflammation. We have not found them to trigger a specific pattern of response that would facilitate their identification. All have, at some point, been reported to cause foreign body giant cell reactions, fibrosis, recurrent hemorrhage, or eosinophilic inflammation.^1^^,^^3^^,^^6^^,^^14^^,^^15^^,^^25–28^ The degree of inflammation likely depends on the amount of material left in situ.^29^ Some nuances exist; for example, flowable hemostatic agents are more prone to lead to pseudocyst formation and anaphylactic reactions occur only with certain agents (eg, collagen-based hemostats).^2^^,^^30^ Identification of various types of hemostatic agents and materials in neurosurgical pathology is relatively straightforward once one is familiar with their characteristic appearances.
